# An Account of the Effects of Mahogany Wood in Cases of Diarrhœa

**Published:** 1795

**Authors:** Francis Hughes

**Affiliations:** Surgeon of the General Infirmary at Stafford.


X. *An Account of the Effects of Mahogany Wood in Cases of Diarrhœa. By Mr.* Francis Hughes, *Surgeon of the General Infirmary at Stafford. Communicated in a Letter to Mr.* John Pearson, *Surgeon of the Lock Hospital, and by him to Dr.* Simmons.

AN accidental circumstance first suggested to me the idea that mahogany wood might prove serviceable as a medicine; for I did not then know that any part of the tree had been employed for medicinal purposes. I was accordingly induced to make use of it in cases of diarrhœa, both in decoction and in the form of an extract; and after repeated trials, I can venture to assert that I have not been disappointed in the expectations I had formed of its efficacy.

For the decoction I boil an ounce of the shavings of Jamaica mahogany wood in two pints of water, till one pint of the liquor is wasted, and then strain off the remainder for use.

The extract I make use of has been prepared by by boiling the ſhavings of the ſame wood in repeated affuſions of freſh water, in the ſame proportion and manner as are directed for the extract of logwood *(extractum hæmatoxyli)* of the London Pharmacopœia. The quantity of extract obtained in this way amounts to ſomething more than $\frac{1}{8}$ of the ſhavings employed. The Honduras mahogany wood is of a paler colour, and leſs aſtringent than the Jamaica, and does not yield quite $\frac{1}{10}$ part of extract.

Both the decoction and extract are very bitter and aſtringent, leaving a roughneſs in the mouth for ſome time after they have been taſted.

The extract, in its appearance, reſembles gum kino. It diſſolves completely in water, and in ſpirit of wine, and ſtrikes a black colour with ſalt of ſteel.

The following are ſome of the Caſes in which I have employed theſe remedies.

### CASE I.

In July, 1793, a ſoldier belonging to a regiment on the Iriſh eſtabliſhment, who is a native of Stafford, was ſent hither from his regiment giment for the recovery of his health. He had for fome time been unfit for duty, and was much reduced by a diarrhœa, which having come on after a fever, had continued feveral months, and refifted a variety of medicines.

I gave him an ounce of the decoction three times a day, and as it fat eafy on his ftomach, and feemed to have a good effect, the dofe, after the third day, was increafed to an ounce and a half. He perfevered in the ufe of it during fixteen days; the diarrhœa gradually fubfided; his appetite and ftrength returned; and at the end of that time he was fufficiently recovered to go back to his regiment in Ireland.

### CASE II.

A woman of a thin, delicate habit, applied to me in October, 1793, on account of a violent diarrhœa, for which fhe had taken different medicines without any good effect. It had come on, fhe faid, after fitting up a whole night in wet clothes, and had continued more than a fortnight; fhe was free from fever.

I directed her to take pills compofed of fix grains grains of the extract, three times a day. Within the ſpace of a week the diarrhœa was much abated, and ſhe had acquired ſtrength; ſhe perſevered, however, in the uſe of the medicine for the ſpace of three weeks, at the end of which time the complaint had entirely ceaſed. A fluor albus, with which ſhe had been troubled many months, was likewiſe much abated; but perhaps this latter circumſtance ought rather to be aſcribed to the improved ſtate of her general health, than to any ſpecific effect of the medicine.

### CASE III.

In January, 1794, I was applied to by a man fifty years old, who for ſeveral years had been a hard drinker, and was now extremely emaciated; his legs were oedematous; he had no appetite; was ſubject to frequent vomiting, and had a ſlight diarrhœa.

I gave him aromatic bitters for ſeveral days, but finding no amendment, I determined to have recourſe to the mahogany. I gave him eight grains of the extract, made into pills, three times a day. At the end of five days his diſpoſition difpofition to vomit had ceafed, and he had a little appetite. He continued the ufe of the medicine for ten days longer, and was then fo much relieved as to be able to walk and ride out every day. This ftate of amendment continued for a fortnight, when he relapfed into his old habit of drinking, and his former fymptoms returned. Recourfe was again had to the fame medicine, but without effect.

To the above I could add many other inftances of the good effects of the extract and decoction in cafes of long continued diarrhœa, where the complaint feemed to depend on a morbid irritability of the ftomach and inteftines, and where the ufe of tonic and aftringent medicines appeared to be indicated. The few hiftories I have related will, I truft, be fufficient to point out the modes of adminiftering the remedies in queftion, and the effects that may be expected from them; and perhaps will induce medical practitioners to extend a trial of their efficacy to other difeafes.

The dofes in which I have hitherto given thefe remedies have been fmall; but much larger dofes may be given with fafety, and in many cafes will, I am perfuaded, be more efficacious.

To

To try the effect of a confiderable dofe on the ftomach, I took two ounces of a decoction, prepared by boiling two ounces of the fhavings in two pints of water to a pint, which is twice the ftrength of the decoction defcribed in Cafe I. and which I have ufually adminiftered. At firft I perceived no effect from it; but at the end of ten minutes a difagreeable naufea came on, with a flight pain at the ftomach, and a glowing fenfation fimilar to that produced by the taking a glafs of ftrong wine. Thefe effects gradually went off in about half an hour, and I felt no other inconvenience from the dofe.

*Stafford, Feb.* 12, 1794.

